# Overexpression of Rice NAC Gene *SNAC1* Improves Drought and Salt Tolerance by Enhancing Root Development and Reducing Transpiration Rate in Transgenic Cotton

**DOI:** 10.1371/journal.pone.0086895

**Published:** 2014-01-28

**Authors:** Guanze Liu, Xuelin Li, Shuangxia Jin, Xuyan Liu, Longfu Zhu, Yichun Nie, Xianlong Zhang

**Affiliations:** 1 National Key Laboratory of Crop Genetic Improvement, Huazhong Agricultural University, Wuhan, Hubei, P. R. China; 2 College of Tobacco Science, Yunnan Agricultural University, Kunming, Yunnan, P. R. China; 3 Agricultural College, Henan University of Science and Technology, Luoyang, Henan, P. R. China; New Mexico State University, United States of America

## Abstract

The *SNAC1* gene belongs to the stress-related NAC superfamily of transcription factors. It was identified from rice and overexpressed in cotton cultivar YZ1 by *Agrobacterium tumefaciens*-mediated transformation. *SNAC1*-overexpressing cotton plants showed more vigorous growth, especially in terms of root development, than the wild-type plants in the presence of 250 mM NaCl under hydroponic growth conditions. The content of proline was enhanced but the MDA content was decreased in the transgenic cotton seedlings under drought and salt treatments compared to the wild-type. Furthermore, *SNAC1*-overexpressing cotton plants also displayed significantly improved tolerance to both drought and salt stresses in the greenhouse. The performances of the *SNAC1*-overexpressing lines under drought and salt stress were significantly better than those of the wild-type in terms of the boll number. During the drought and salt treatments, the transpiration rate of transgenic plants significantly decreased in comparison to the wild-type, but the photosynthesis rate maintained the same at the flowering stage in the transgenic plants. These results suggested that overexpression of *SNAC1* improve more tolerance to drought and salt in cotton through enhanced root development and reduced transpiration rates.

## Introduction

Abiotic stresses such as drought, salinity and extreme temperatures have a crucial impact on agricultural productivity and yields. Climate models indicated that abiotic stresses would increase in the near future because of global climate change [1]. Therefore, drought and salinity would be the two major factors that adversely affect crop growth and productivity. Cotton (*Gossypium hirsutum L*.) is an important commercial crop worldwide, and used as a source of fiber and edible oil. Cotton showed higher drought and salt tolerance than other major crops such as rice, wheat and maize. Although that, abiotic stress still greatly affected cotton in growth and yield. Farmers plan to enlarge the planted area of cotton in western China, which is not suitable for food crops because of salinity and water shortage. Therefore, improved drought and salt tolerance of cotton through biotechnology has become an urgent task.

The NAC transcription factors have been characterized for their roles in plant growth, development, and stress tolerance. NAC was originally derived from the names of the first three proteins containing NAM (no apical meristem), ATAF1-2 and CUC2 (cup-shaped cotyledon), that contain a similar DNA-binding domain. NAC proteins appeared to be widespread in plants such as Arabidopsis, rice, wheat, soya bean and cotton [2], [3], [4], [5], and comprised a large plant-specific family, which included 110 members in Arabidopsis and 140 members in rice, and only a few had been identified with diverse functions in plants [6]. Recently, Several studies reported that a subfamily of NAC transcription factors played a pivotal role in various abiotic stresses including salinity, drought and low temperature [4], [7], [8]. Previous works indicated that the overexpression of *ANAC019*, *ANAC055*, and *ANAC072* caused increased drought tolerance in transgenic plants through changing the transcription of a limited number of non-specific salt- and drought-responsive genes [9], [10]. The overexpression of *SNAC1* in rice was another important example, which enhanced salt and drought tolerance and grain yield in the field test [11]. Recent results showed that overexpression of *OsNAC5* significantly enlarged roots and enhanced drought tolerance and grain yield under field conditions, as well [12]. At present, improved drought and salt tolerance could also be achieved by the overexpression of diverse NAC factors in species ranging from Arabidopsis, rice, chickpea, wheat, and tomato [4], [13], [14], [15], [16]. Constitutive overexpression of NAC gene in transgenic plants occasionally led to detrimental consequences such as dwarfing, late flowering and lower seed yield, which could be overcomed by preferentially employed stress-inducible or tissue-specific promoters such as *OsNAC6* or *RCc3* [12], [17]. Thus, the research gave the evident functional of stress-responsive NAC genes, a meticulous characterization of their response to abiotic stress is crucial in conferring broad stress tolerance to plants.

A previous study suggested that *SNAC1*, which encoded an NAC transcription factor, was predominantly induced in guard cells by drought in rice. Further results showed that *SNAC1*-overexpression in rice significantly improved drought tolerance under field conditions and strong tolerance to salt stress. The increased drought tolerance might be partly explained by the reduced transpiration rate and an increased sensitivity to ABA. In addition, *SNAC1* overexpression did not result in unwanted dwarf phenotype in transgenic rice [11]. It was also important to be considered that the new transgenic lines with acquired stress-resistance phenotypes did not cause yield penalty and/or growth retardation. And breeders might be able to use *SNAC1* to increase stress tolerance by employing biotechnology in rice [18]. It was unclear whether *SNAC1* could be employed in other crops to improve tolerance to abiotic stress.

We reported that ectopic *SNAC1* expression in cotton led to improved drought and salt tolerance at the vegetative and reproductive stages in transgenic plants. Our results also showed that *SNAC1-*overexpression significantly enlarged root biomass and increased the boll number during drought and salt treatment in the greenhouse. This findings implied that *SNAC1* in improving agronomic traits and economic characteristics of cotton by ectopic expression would be an efficient way to accelerate the cotton breeding program.

## Materials and Methods

### Vector Construction and Cotton Transformation

**Figure 1:**
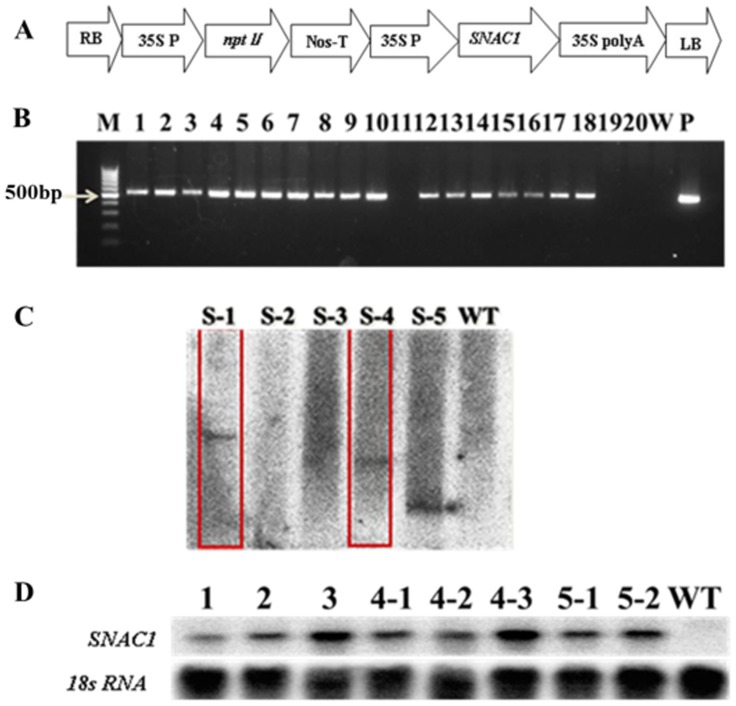
Overexpression vector and molecular identification of transgenic plants. (A): Map of SNAC1-overexpressing vector with the *npt II* gene as a screening marker. (B): Molecular analysis of *SNAC1* transgenic plants. M: Marker; 1–20: T_1_ generation of transgenic plants (1–4 from S-1, 5–8 from S-2, 9–12 from S-3, 13–16 from S-4, 17–20 from S-5); W: Wild-type plant; P: Plasmid; (C): Southern blotting analysis of transgenic plants. S-1: *SNAC1* transgenic line-1; S-2: *SNAC1* transgenic line-2; S-3: *SNAC1* transgenic line-3; S-4: *SNAC1* transgenic line-4; S-5: *SNAC1* transgenic line-5; WT: wild-type plant. (**D**) Northern blot analysis of *SNAC1*-overexpressing transgenic plants; 1: Transgenic plant from line S-1; 2: Transgenic plant from line S-2; 3: Transgenic plant from line S-3; 4-1, 4-2, 4-3: Three individual transgenic plants from line S-4; 5-1, 5-2: Two individual transgenic plants from line S-5; WT: Wild-type plant. Red box: The selected lines.

The full-length of *SNAC1* from rice was kindly donated by Professor Lizhong Xiong (Huazhong Agricultural University). The coding sequence was ligated into a site within the binary vector pCAMBIA2300S that contains the neomycin phosphotransferase gene, or *NPT II*, which was used as the selective marker [19]. The overexpression vector (Figure 1A) was then introduced into *Agrobacterium tumefaciens* strain LBA4404, which was used to transform cotton according to an established method [20]. Segments of hypocotyls from *G. hirsutum cv* YZ1 were used as explants for transformation. The callus induction and plant regeneration protocols were previously described by Jin et al. [21].

### DNA Isolation and Southern Blot Analysis

Genomic DNA was isolated from the young leaves of transgenic and wild-type plants using a Plant Genomic DNA Kit (Tiangen Biotech, China). The PCR amplification was carried out with the specific primers of the *SNAC1* gene. Sequences of the forward primer and the reverse primer were 5′-AGCGAGAAGCAAGCAAGA AGCG-3′and 5′-ACAGCACCCAATCATCCAACCT-3′, respectively. The predicted PCR product was 509 bp in length. For Southern hybridization, cotton genomic DNA was digested with *Hind III*, electrophoresed on 0.8% agarose gel and blotted onto a Hybond N^+^ membrane for hybridization [22]. The fragment of *SNAC1* gene from PCR reaction mentioned above was used as a gene-specific probe and prepared using the Prime-a-Gene Labeling System (Promega, USA). After hybridization and stringent washing, the radioactive membranes were exposed to an imaging plate (Fuji Photo Film, Japan) for 5 h or overnight to record the image.

### Total RNA Isolation for Northern Blot Analysis

Total RNA was extracted from the young leaves of transgenic and wild-type cotton plants [22]. For the northern blot analysis, 10 µg of total RNA was separated on a 1.2% formaldehyde-containing agarose gel. After electrophoresis, the gel was rinsed with DEPC-H_2_O, equilibrated with 20× SSC and then transferred to a Hybond N^+^ nylon membrane by capillary blotting. The membranes were air dried and baked for 2 h at 80°C in an oven. Hybridization was performed at 65°C with a *SNAC1* probe. The same blot was then stripped and reprobed with an 18s RNA probe. The washing and imaging steps were the same as for the Southern blot procedure.

### Hydroponic Cotton Growth under High-salt Conditions

Cotton seeds from wild-type and transgenic lines were sterilized and germinated on sterile Stewart’s germination media [23]. Transgenic seeds were grown on a medium containing 50 mg L^−1^ kanamycin to screen the transgenic plants. Positive transgenic plants with complete rooting systems, including lateral roots, were used for this experiments. At the two-leaf stage, wild-type and transgenic seedlings were transferred to Hoagland solution. After 21 days at normal conditions, six seedlings of each replicate from both wild-type and transgenic lines were exposed to different salinity conditions in which NaCl was added to the Hoagland solution at 50, 100, 150, and 200 mM increments every 72 h until a final concentration of 250 mM was reached and maintained for one week. All wild-type and transgenic lines were then removed from the tubs and dried with paper towels to measure the fresh shoot and fresh root weights. The total plant dry weights were calculated for a biomass determination by drying the samples for 72 h in an oven at 80°C.

### Proline and Malondialdehyde (MDA) Content Measurements

Cotton seeds from wild-type and transgenic lines were sterilized and germinated on sterile Stewart’s germination media to screen the transgenic plants as described. Each plant was transferred to a small pot of uniform soil mix and then placed in a larger tub container. Every tub container included fifteen plants (each tub had five wild-type, five transgenic line S-1 and five S-4 plants). The eighteen-day-old plants were grown in a greenhouse and then subjected to drought and salt stress. For the control conditions, every tub container received 4000 ml of water. For drought stress, the plants were subjected to water stress by withholding half the water for two weeks. For salt stress, plants were watered with 250 mM NaCl solution for two weeks. The plants’ proline and MDA contents were recorded. The levels of proline content in leaf tissue were determined according to the method outlined in reference [24]. Samples weighing approximately 100 mg were collected from the first fully expanded leaf of stressed (drought-stress and salt-stress) or control plants, and they were then placed in 10 ml microcentrifuge tubes containing 3 ml of 3% sulfosalicylic acid for grinding. Ground samples were centrifuged, and 1 ml of the supernatant was mixed with 1 ml of acid ninhydrin solution (1.25 g ninhydrin in 30 ml of glacial acetic acid and 20 ml of 6 M phosphoric acid) and 1 ml of glacial acetic acid in a fresh glass tube. The tubes were capped and incubated at 100°C in a water bath for 30 min. The reaction was terminated by chilling the tubes on ice. Then, 2 ml of toluene was added to each tube, and the mixture was vortexed for 10 s. One milliliter of the upper toluene phase containing the chromophore was aspirated and read at 520 nm in a quartz cuvette. The proline concentrations were estimated based on a standard curve for proline.

In addition, the same cotton seedling was used to analyze leaf lipid peroxidation by estimating the formation of malondialdehyde (MDA), an end product of lipid peroxidation. A 100 mg leaf piece was collected from the first fully expanded leaf (excluding the midrib) and homogenized in 5 ml of 10% TCA (trichloroacetic acid). The extract was centrifuged at 10,000 rpm for 10 min. The reaction was initiated by adding 2 ml of supernatant to 2 ml of 0.6% TBA (thiobarbituric acid dissolved in 10% TCA), and the mixture was then incubated at room temperature for 2 h. The reaction mixture was boiled at 100°C for 1 h. After cooling to room temperature, the OD was measured at 450 nm, 532 nm and 600 nm. The MDA content was estimated using the formula reported in citation [25].

### Plant Performance under Salt and Drought Treatment in the Greenhouse

Cotton seeds from the wild-type and two transgenic lines were planted in soil mix. These plants were allowed to grow under normal conditions until the budding stage, and uniform plants were then divided into three groups: a well-watered control group, a drought stress group and a salt stress group. Each group consisted of eighteen plants. Stress treatment was applied at the budding and flowering stages. During the budding stage, the well-watered plants were irrigated with 2000 ml of water every ten days. For drought stress, plants were irrigated with 1000 ml of water per pot every ten days for 20 days. For the salt stress group, plants were irrigated with 2000 ml of 100 mM NaCl solution every ten days for 20 days. After 20 days of treatment, the photosynthesis, stomatal conductance and transpiration rate were measured using a portable photosynthesis system (Li-6400XT, LI-COR Inc, Lincoln, NE, USA). The photosynthesis, stomatal conductance and transpiration rate were assessed at a CO_2_ concentration of 400 µmol mol^−1^, a relative humidity of 50%, a chamber temperature of 28°C, an air flow of 500 µmol s^−1^ and a photon flux density of 1500 µmol m^−2^s^−1^. The instrument was stabilized according to the manufacturer’s guidelines [26]. Steady-state levels of reference CO_2_ and H_2_O were observed before taking the measurements. When all the plants were nearly flowering, the second treatment was applied. During the flowering stage, the well-watered plants were irrigated with 2000 ml of water every five days. For drought treatment, irrigation was reduced to 1000 ml of water per pot every ten days. For salt treatment, the NaCl concentration was increased gradually to 250 mM and irrigated with 2000 ml of NaCl solution every five days. After 20 days of treatment, the photosynthesis, stomatal conductance and transpiration rate were measured as described earlier. When the flowering stage stress was finished, the plants were re-watered with 3000 ml of water per pot, and the biomass analysis was carried out after one week. All stress experiments were conducted in a greenhouse at 35±3°C/28±3°C (day/night) with a relative humidity of 50–70% and a photoperiod of 14/10 h (light/dark).

### Rate of Water Loss from Excised Leaves

The water loss from detached leaves of wild-type and transgenic was measured by monitoring the fresh weight loss at the indicated time points.

## Results

### Transformation and Molecular Analyses of *SNAC1*-overexpressing Cotton Plants

The pCAMBIA2300S*-SNAC1* construct was introduced into cotton via *Agrobacterium*-mediated transformation, and 5 independent transgenic lines were produced. The T1 population (progeny of T0 plants) was then employed for molecular analysis. The presence and integrity of the transgene were confirmed using PCR analysis on the genomic DNA with specific primers for *SNAC1* (Figure 1B). The results of southern blot showed three transgenic lines that appeared to contain a single-copy insertion, and they were identified as S-1, S-4 and S-5 (Figure 1C). Transgenic lines S-2 and S-3 had been proved as positive transgenic plants through PCR analysis, but did not show hybridization signals in southern blotting analysis. We deduced that the reason might due to the poor quality of DNA from transgenic plant, which influenced the efficiency of membrane blotting (Figure 1B, C). To check the expression level of *SNAC1*, northern blotting was employed with the T2 transgenic cotton lines. The results showed that mRNA transcription level of *SNAC1* was diverse among the five transgenic lines (Figure 1D). Two independent lines, S-1 and S-4, with high expression level of *SNAC1*, were selected and used for drought and salt-tolerance analysis.

### *SNAC1-*overexpression Increases Tolerance to Salinity under Hydroponic Conditions in Cotton

**Figure 2:**
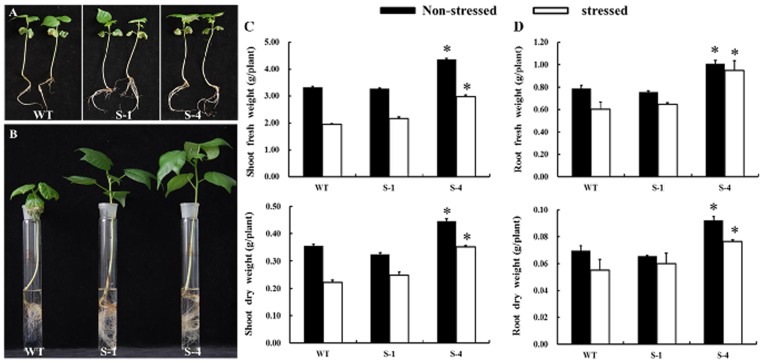
Salt treatment of *SNAC1*-overexpressing plants under hydroponic conditions. (A): Phenotype of wild-type and *SNAC1*-overexpressing transgenic plants before salt treatment; (B): Phenotype of wild-type and *SNAC1*-overexpressing transgenic plants after salt stress with 250 mmol/L NaCl for 1 week. (C, D): Biomass analysis of wild-type and *SNAC1*-overexpressing transgenic plants under normal conditions and with salt treatment; WT: wild-type plant; S-1: *SNAC1*-overexpressing transgenic plant from transgenic line S-1; S-4: *SNAC1*-overexpressing transgenic plant from transgenic line S-4. *statistically significant at 5%.

To identify whether *SNAC1*-overexpression enhances salt tolerance in transgenic cotton, we performed hydroponic experiments in a greenhouse. Transgenic lines S-1, S-4 and wild-type plants were grown hydroponically in a saline medium and under normal conditions. No obvious phenotype differences were found between the wild-type and transgenic plants before salt treatment (Figure 2A). Uniform seedlings of transgenic lines S-1 and S-4 and wild-type plants were cultured in hydroponic solution with NaCl concentrations from 50 to 250 mM, with a 50 mM addition every 72 hrs. After 7 days under the treatment with 250 mM NaCl, the wild-type plants exhibited growth retardation and leaf wilting, but the transgenic line grew better than the wild-type plants (Figure 2B). Moreover, transgenic lines S-1 and S-4 had considerably larger shoots and roots than those of the wild-type plants (Figure 2B), particularly transgenic line S-4, which produced significantly more shoot and root biomass than the wild-type plants (Figure 2C, D). The S-1 and S-4 lines produced 12% and 59% more dry shoot mass and 9% and 38% more dry root mass, respectively, compared to the wild-type plants under the salt treatment. All the plants were also measured under controlled conditions. Interestingly, the transgenic plants of line S-4 had increased biomass production under normal conditions (Figure 2C, D).

### Overexpressing *SNAC1* Elevated Osmoprotectant Levels in the Transgenic Seedlings

**Figure 3:**
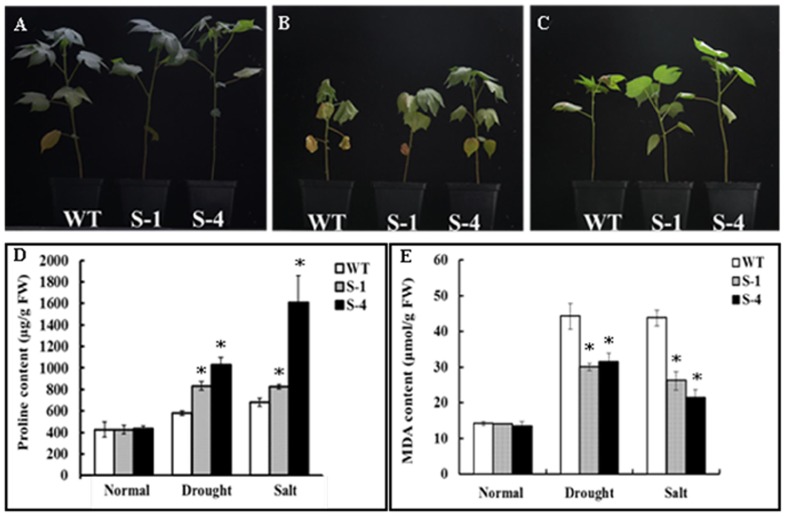
Detection of stress tolerance in *SNAC1*-overexpression transgenic plants at the seedling stage. (A): Phenotype of wild-type and *SNAC1*-overexpressing transgenic plants under normal conditions; (B): Phenotype of wild-type and *SNAC1*-overexpressing transgenic plants after two weeks of drought stress; (C): Phenotype of wild-type and *SNAC1*-overexpressing transgenic plants after two weeks of salt stress with a 250 mmol/L NaCl solution; (D): Proline content analysis under normal conditions and stress treatments; (E): MDA content analysis under normal conditions and stress treatments; WT: wild-type plant; S-1: *SNAC1*-overexpression transgenic plant from transgenic line S-1; S-4: *SNAC1*-overexpression transgenic plant from transgenic line S-4. *statistically significant at 5%.

*SNAC1*-overexpressing and wild-type plants were grown under drought and salt treatment conditions to examine stress tolerance in soil. At two-leaf stage, each plant was transplanted into a large plastic container containing fifteen pots with five pots for each line and one plant in every pot. All the plants of transgenic lines S-1, S-4 and wild-type plants were divided into three groups including normal, drought and salt treatments. All of the wild-type and transgenic plants grew well under normal conditions. While, the leaves of the wild-type plants withered more significantly than the transgenic lines after two weeks treatment of drought stress. The leaves of S-1 experienced medium wilting, and the leaves of S-4 showed slight wilting (Figure 3A, B). Under salt treatment, transgenic lines S-1 and S-4 shrank slightly, but the wild-type plants clearly withered and had shrunken leaves (Figure 3C). Meanwhile, we also estimated the levels of osmoprotectants such as proline and MDA in the leaves of wild-type and transgenic plants in response to different treatments. No significantly different in proline content was observed between the wild-type and transgenic plants under normal conditions. Under the drought and salt treatments, the content of proline was elevated in all of the plants, but it was remarkedly higher in two transgenic lines. Under drought treatment, the proline content increased up to 1.4-fold and 1.8-fold in the S-1 and S-4 transgenic lines, respectively, which was significantly higher than that of the wild-type plants. Similar results were also found in the two transgenic lines with 1.2-fold and 2.4-fold in proline content, which was significantly higher than that in the wild-type plants, after 250 mM NaCl treatment (Figure 3D).

A remarkable high level of MDA was detected in the wild-type and transgenic plants after drought and salt treatment when compared with the normal condition plants. A maximum of 3.1 times in the MDA level was observed in wild-type plants, but an increase of 2.1-fold for S-1 and 2.3-fold for S-4 in the MDA content was measured under drought stress. The MDA levels were also significantly increased under salt treatment in all seedlings, while the amounts of MDA in lines S-1 and S-4 were remarkablely less than that in the wild-type (Figure 3E). There were no significant differences in the MDA levels between wild-type and transgenic plants under normal conditions (Figure 3E). These results indicated that *SNAC1-*overexpression in cotton could alleviate cell membrane injury under both drought and salt stresses.

### *SNAC1*-overexpression Increases Cotton Tolerance to Drought and Salt Stress in the Greenhouse

**Figure 4:**
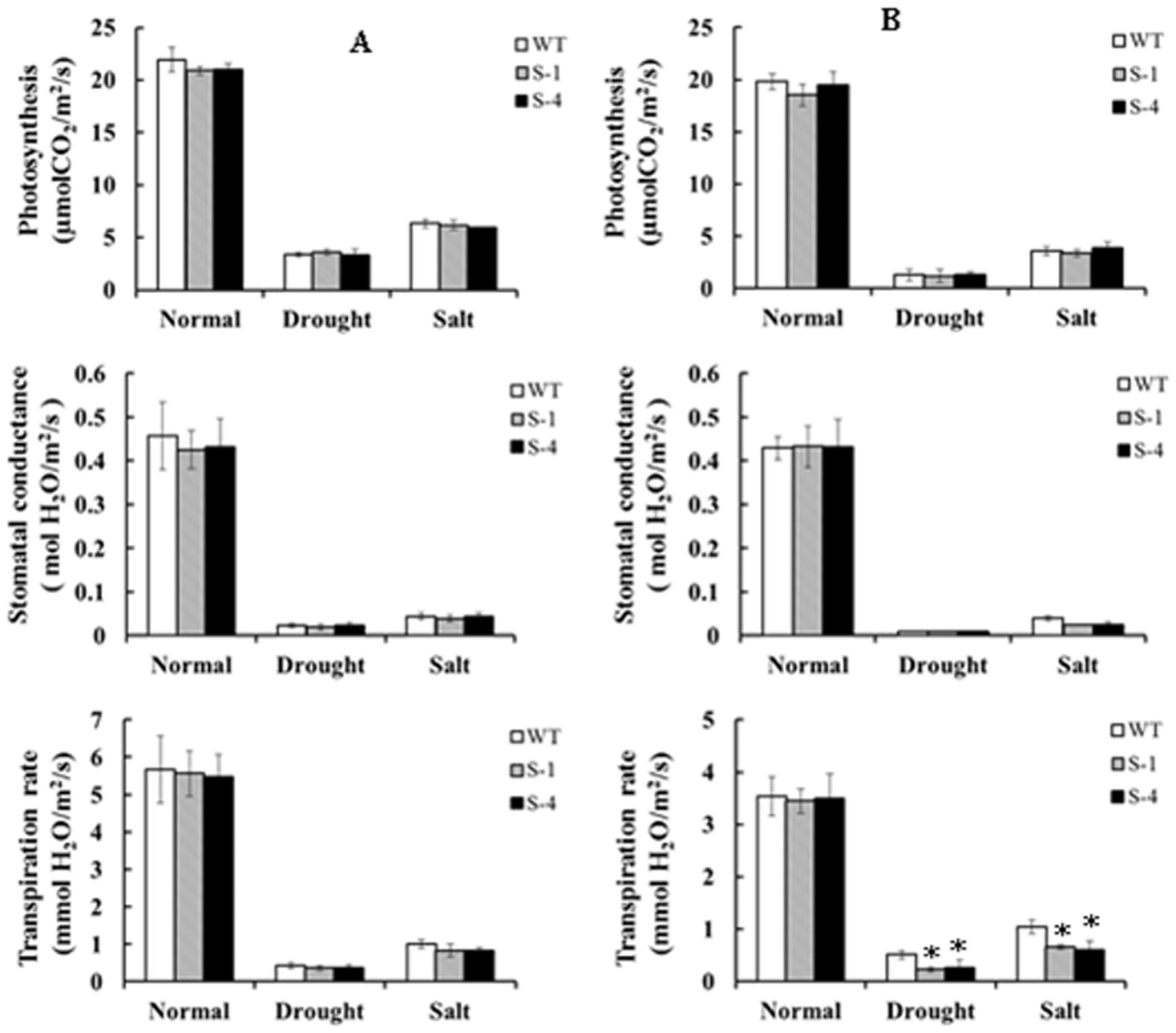
Photosynthetic performance of wild-type and *SNAC1*-overexpressing transgenic plants at different stages under stress treatment. (A): Photosynthetic performance of wild-type and *SNAC1*-overexpressing transgenic plant after 20 days of drought stress or salt stress under 100 mmol/L NaCl solution during the budding stage; (B): Photosynthetic performance of wild-type and *SNAC1*-overexpressing transgenic plants after 20 days of drought stress or salt stress in 250 mmol/L NaCl solution during the flowering stage; WT: wild-type plant; S-1: *SNAC1*-overexpressing transgenic plant from transgenic line S-1; S-4: *SNAC1*-overexpressing transgenic plant from transgenic line S-4. *statistically significant at 5%.

**Figure 5:**
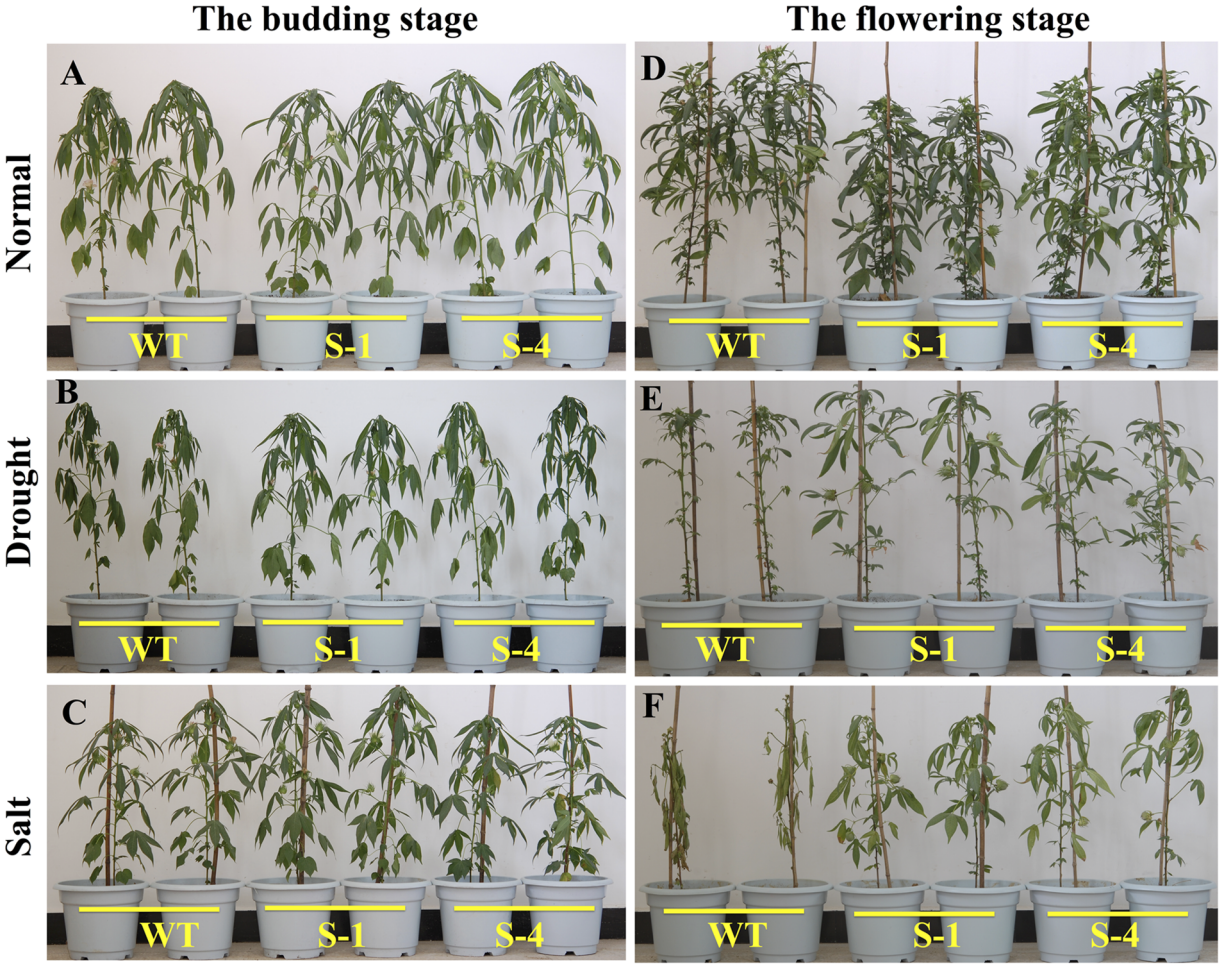
Phenotype of *SNAC1* transgenic and wild-type plants at budding and flowering stages during stress treatments. (A), (B), (C): Phenotype of wild-type and *SNAC1*-overexpressing transgenic plants under stress at the budding stage; (A): normal conditions after 20 d; (B): drought stress after 20 d; (C): 100 mmol/L NaCl stress after 20 d; (D),(E),(F): Phenotype of wild-type and *SNAC1*-overexpressing transgenic plants under stress treatments at the flowering stage; (D): normal conditions after 20 d at the flowering stage; (E): Drought stress after 20 d at the flowering stage; (F): Salt stress in 250 mmol/L NaCl solution for 20 d at the flowering stage. WT: Wild-type; S-1: *SNAC1* plants from transgenic line S-1; S-4: *SNAC1* plants from transgenic line S-4.

To evaluate the drought and salt tolerance of transgenic cotton at the budding and flowering stages, transgenic and wild-type plants were subjected to drought and salt stress in greenhouse. The results showed that no significant difference was found in the photosynthesis, stomatal conductance, and transpiration rate between the transgenic and wild-type plants during the budding stage under both treatments (Figure 4A). Although photosynthesis, stomatal conductance and transpiration rate decreased rapidly in all plants under the drought and salt stress compared to normal conditions, the transgenic and wild-type plants did not show significant phenotype differences at the budding stage under different conditions (Figure 5A, B, C). When the plants were subjected to stress for twenty days at the flowering stage, the photosynthesis and stomatal conductance values decreased greatly under drought or salt stress treatments in all plants. While, no difference was detected between the wild-type and transgenic plants. However, the transpiration rate in transgenic plants significantly decreased compared to the wild-type plants under drought and salt treatments (Figure 4B), which might be inferred that overexpression of *SNAC1* in cotton could improve efficiency in water usage. In conclusion, the transgenic plants had a higher tolerance than the wild-type at the reproductive stage under both stresses (Figure 5D, E, F).

**Figure 6:**
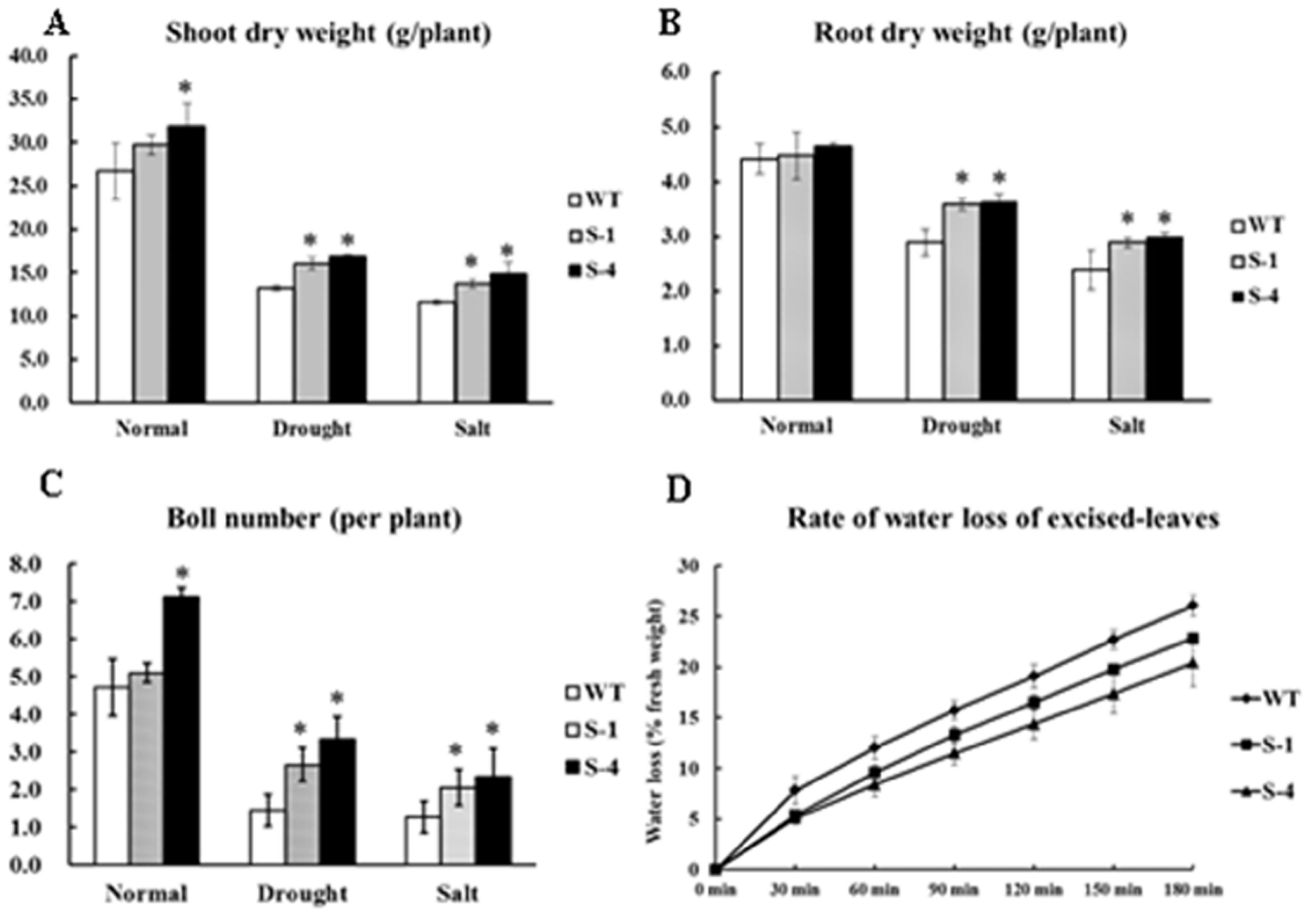
Biomass analysis of wild-type and *SNAC1*-overexpressing transgenic plants under normal condition and stress treatments. (A): Shoot dry weight analysis of wild-type and *SNAC1* plants after different stress treatments; (B): Root dry weight analysis of wild-type and *SNAC1* plants after different stress treatments; (C): Final boll number of plant under different stress treatment in the greenhouse; (D): Rate of water loss from excised leaves. WT: wild-type plants; S-1: *SNAC1* plants from transgenic line S-1; S-4: *SNAC1*-overexpression plant from transgenic line S-4. *statistically significant at 5%.

**Figure 7:**
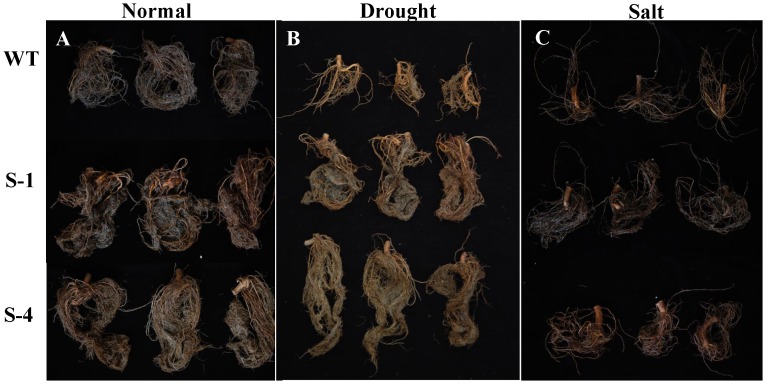
The root growth phenotype of wild-type and *SNAC1*-overexpressing transgenic plants after normal condition and stress treatments. (A): Normal conditions of wide-type and *SNAC1* plants; (B): Drought stress of wide-type and *SNAC1* plants; (C): Salt stress of wide-type and *SNAC1* plant. WT: Wild-type; S-1: *SNAC1* plants from transgenic line S-1; S-4: *SNAC1* plants from transgenic line S-4.

Furthermore, the biomass of the plants was calculated for all treatments. The transgenic cotton produced significantly more shoots and roots under the stress treatments (Figure 6A, B). When subjected to drought, lines S-1 and S-4 produced 22% and 23% more dry shoot mass and 24% and 26% more dry root mass than the wild-type plants, respectively. Under the salt treatment, transgenic lines S-1 and S-4 had 18% and 28% more dry shoot mass and 21% and 26% more dry root mass, respectively. In addition, the total boll number was also calculated from these plants and the results showed that 85% and 131% more bolls under drought stress and 61% and 83% more bolls under salt stress were produced in lines S-1 and S-4 than the wild-type plants, respectively (Figure 6C). The rate of water loss (RWL) from excised leaves was also monitored at the indicated time points. The RWL of wild-type plants increased to 26%, but only 20% and 22% were found in S-1 and S-4 (Figure 6D). The result suggested that the water-holding ability of transgenic plant was higher than that of wild-type plants. And the transgenic plants showed more roots than wide-type plants under normal conditions and stress treatments (Figure 7). Generally, overexpression of *SNAC1* in cotton significantly improved abiotic stress tolerance with increased biomass and boll numbers under reduced irrigation and salinity conditions.

## Discussion

One major challenge in modern agricultural production is the growing demand for food paralleled by dramatic losses of arable land as a consequence of increasingly severe soil destruction by abiotic environmental conditions [27]. Transcription factors played important roles in the regulation of gene expression in response to abiotic stresses and could be employed as candidate genes for the genetic engineering to improve stress tolerance in crops [18]. By increasing the understanding of the NAC transcription factor class in controlling abiotic stress responses, practical approaches had been developed for engineering stress tolerance in crops [27]. *SNAC1* was predominantly induced in guard cells by drought, and overexpression of *SNAC1* in rice showed significantly improved drought resistance and salt tolerance with higher seed setting than the control under severe drought stress conditions at the reproductive stage [11].

As expected, *SNAC1*-overexpressing cotton plants performed better than wild-type plants under drought and salinity conditions at the vegetative and reproductive stages. More importantly, transgenic *SNAC1* cotton showed no obvious differences from the wild-type plants in all investigated traits under normal condition, which in accordance with the results in transgenic rice. In our study, transgenic cotton grew much better than wide-type plants in the presence of 250 mM NaCl under hydroponic growth conditions. The increased salinity tolerance was measured by quantifying the biomass of cotton plants. The dry shoot and dry root masses of all *SNAC1*-overexpressing seedlings were significantly higher than those of wild-type plants grown under 250 mM NaCl conditions. In particular, the dry root masses of transgenic S-4 seedlings were higher than those of wild-type seedlings. The soil-grown *SNAC1*-overexpressing cotton also displayed significantly improved salt and drought tolerance in the greenhouse. The dry biomasses of both the shoots and roots of the *SNAC1*-overexpressing plants were significantly higher than those of the wide-type plants. In rice, *SNAC1*-overexpressing plants were no different from wild-type plants in terms of root depth and volume. Therefore, it was possible that this gene had a very limited contribution to root development. Recently, *OsNAC9* (which is identical to the *SNAC1* gene) was overexpressed under the control of root-specific promoter *RCc3* enhanced drought resistance in rice, and increased root diameters were found in the transgenic plants [28]. In a previous study, 18 NAC domain factors were identified from expression profiling of stress-treated rice. *OsNAC10* and *SNAC1/OsNAC9*, which were members of subgroup I, were believed to have similar stress response function because of their sequence similarity. *OsNAC10-*overexpressing in rice caused enlarged roots, enhanced drought tolerance, and significantly increased grain yield under field drought conditions [29]. These results suggested the *SNAC1* may confer drought resistance through the altered root architecture. A longer root system should have facilitated water absorption from deeper soils, and thus strengthened drought tolerance and increased biomass under water-deficit conditions [30]. In our study, the root biomass of transgenic lines was higher than that of the wild-typ whether in normal condition or stress. The overexpression of *SNAC1* in cotton significantly increased root development, which suggested that the development of larger roots should be favorable for drought resistance breeding.

In a recent report, *OsSRO1c* was characterized as a direct target of SNAC1 [31]. Moreover, *SNAC1*-overexpressing and *OsSRO1c*-overexpressing plants both increased stomatal closure and reduced water loss under drought stress [11], [31]. In our study, photosynthesis was not significantly affected in the transgenic plants in relative to the wild-type under different conditions. While, the transpiration rate was lower in the transgenic plants than the wild-type under drought and salt treatments. Further investigation of the RWL from excised leaves in transgenic and wild-type plants showed that transgenic leaves had less water loss. These results implied that the enhanced tolerance of *SNAC1*-overexpressing plants was related to water usage efficiency.

Osmotic adjustment is a fundamental cell tolerance response to osmotic stress and could be realized by the accumulation of osmoprotectants. The osmotic potential is a direct reflection of the osmotic adjustment capability at the physiological level and has been used as an effective index for assessing crop genotypes for osmotic stress tolerance [32]. Numerous studies had shown that free proline was the most widely distributed multifunctional osmolyte in many organisms, and it played important roles in enhancing osmotic stress tolerance [33]. In our study, the proline content increased more significantly in transgenic plant than in the wild-type plant under drought and salt stress. In addition, transgenic cotton maintained less MDA in content than wild-type plants during stress treatment, which was in accordance with the improved tolerance to stress in transgenic cotton.

In summary, *SNAC1*-overexpressing cotton plants significantly enhanced drought resistance and salinity tolerance at the vegetative and reproductive stages. The resuls suggested that this gene may show great promise for the genetic improvement of stress tolerance in cotton.
